# Knowledge-Enhanced Compressed Measurements for Detection of Frequency-Hopping Spread Spectrum Signals Based on Task-Specific Information and Deep Neural Networks

**DOI:** 10.3390/e25010011

**Published:** 2022-12-21

**Authors:** Feng Liu, Yinghai Jiang

**Affiliations:** 1 College of Electronic Information and Optical Engineering, Nankai University, Tianjin 300350, China; 2 Tianjin Key Laboratory of Optoelectronic Sensor and Sensing Network Technology, Nankai University, Tianjin 300350, China

**Keywords:** FHSS, knowledge-enhanced compressed measurement, signal detection, task-specific information, deep neural network

## Abstract

The frequency-hopping spread spectrum (FHSS) technique is widely used in secure communications. In this technique, the signal carrier frequency hops over a large band. The conventional non-compressed receiver must sample the signal at high rates to catch the entire frequency-hopping range, which is unfeasible for wide frequency-hopping ranges. In this paper, we propose an efficient adaptive compressed method to measure and detect the FHSS signals non-cooperatively. In contrast to the literature, the FHSS signal-detection method proposed in this paper is achieved directly with compressed sampling rates. The measurement kernels (the non-zero coefficients in the measurement matrix) are designed adaptively, using continuously updated knowledge from the compressed measurement. More importantly, in contrast to the iterative optimizations of the measurement matrices in the literature, the deep neural networks are trained once using task-specific information optimization and repeatedly implemented for measurement kernel design, enabling efficient adaptive detection of the FHSS signals. Simulations show that the proposed method provides stably low missing detection rates, compared to the compressed detection with random measurement kernels and the recently proposed method. Meanwhile, the measurement design in the proposed method is shown to provide improved efficiency, compared to the commonly used recursive method.

## 1. Introduction

In both military and civilian secure communications, the spread spectrum (SS) techniques have been widely used [1]. In these techniques, the spectra of base-band signals are spread into a much wider band. Thus, the SS signals are more resistant to interference or jamming, as common interference or jamming signals can only affect a small fraction of the spread spectrum. Moreover, to catch the entire spread spectrum, the conventional non-cooperative receivers must operate at a high sampling rate. This, in turn, makes the SS signals resistant to non-cooperative detection or interception. The frequency-hopping spread spectrum (FHSS) is one of the most used SS techniques. In the FHSS, the carrier frequency of the signal altered rapidly in a pseudo-random manner, so that the base-band signal is spread into the frequency-hopping range.

The non-cooperative detection of the FHSS signal is the first step of the entire signal interception procedure [2]. Although various methods has been rendered since the 1990s (e.g., methods based on time-frequency analysis [3,4,5,6,7,8], wavelet analysis [4,9,10,11,12,13], auto-correlation analysis [9,14,15,16], likelihood analysis [17,18,19,20,21], etc.), energy thresholding is the most commonly used in FHSS signal detection [22,23,24]. To reduce sampling rate while observing the entire frequency-hopping range, some strategies, such as the channelized filter bank [23,25] and the sweeping spectrum analyzer [20,26,27,28,29], were rendered by dividing the entire spectrum into subbands and observing the individual subbands with relatively lower sampling rates. The signal is claimed to be detected if the energy is higher than the threshold in at least one observed subband. However, as the signals only fall in a relatively narrow band at any given observation time in this scenario, most of the measurements are normally done to background noises, leading to relatively high false-alarm rates.

In 2006, the theory of compressed sensing (CS) was rendered [30,31]. The CS theory states that a signal can be recovered from sub-Nyquist samples with overwhelmed probability, if it can be sparsely represented based on a transform or overcomplete dictionary. In most of the existing literature on the CS-based signal detections, the sparse representations of the signals were exploited, where the dictionaries were assumed to be known [32,33,34,35] or established based on the signal properties. However, an intermediate step of signal reconstruction [36,37] was also proposed in some of these methods. Other detection methods from compressed measurements assume the precise knowledge of the signal expressions. More recently, Liu et al., proposed a method to directly detect the FHSS signals from compressed samples [38,39]. Besides the random measurement kernels (i.e., the non-zero coefficients in the measurement matrix) used in most of the CS literature, a strategy to design the measurement matrix prior to the measurements was also rendered with improved detection performance. However, the measurement kernel design was made based on expensive recursive optimizations of Shannon information. Therefore, it was not practical for online adaptive implementations, as a quick response is usually required. Later, the information optimized compressed measurements [40] and information-based pattern recognition of FHSS signals [41] were also studied by researchers. In 2020, Wang et al., proposed a partial discrete Fourier transform (DFT)-based method to design the measurement matrix for FHSS signal detection, where the mutual information between the signals and the measurements was also considered [42]. However, the measurement matrix was designed prior to the measurement process. Therefore, the measurements could not be adjusted adaptively according to the posterior knowledge of the signals. In addition, the signal samples or priori knowledge of the communication protocol are also required in the training the measurement matrices in that method.

To efficiently extract the key features from the signals, fast signal feature extraction methods were studied over decades. Although methods such as principal component analysis, linear component analysis, independent component analysis, supporting vector machine, etc. were studied in various scenarios, the logics that can be represented within such methods were constrained. In the 1970s, artificial neural networks (ANNs) were proposed to model the logics between the input data and their features or processed results. The ANN was originally rendered to solve the signal classification problem. The model optimizations were done using the training procedure. In recent decades, with the development of the parallel computing and the graphic processing unit (GPU) techniques, the deep neural networks (DNNs) are enabled and is now adopted in various areas in signal processing, such as parameter estimations [43], audio and image encoding [44,45], etc. Efforts were also devoted on the study of the robust DNN training [46]. Recently, the DNNs were also proposed to estimate the parameters of the FHSS signals [47,48]. However, as a common problem with these methods, there is a lack of adaptivity in their implementations.

In this paper, we propose an efficient and adaptive method to detect the FHSS signals non-cooperatively. The adaptivity of this method is achieved with the fusion of the posterior knowledge enhancement algorithm and the DNNs. The posterior knowledge is gained with the gradually increased task-specific information (TSI) [49] in the detection task, while the DNNs are trained to adaptively design the measurement kernels given the updated knowledge of the measured signal. Our proposed method includes several novel contributions:

(1) The FHSS signal-detection method proposed in this paper is achieved directly from the low-rate sampling results without the reconstruction of the original signal.
(2) The quantitative Shannon information is analyzed based on the posterior information of the channel output and is used in the measurement kernel design for the following measurements, which ensures improvement in the FHSS signal-detection accuracy.
(3) More importantly, with an effective combination of the TSI optimization theory and the DNNs in this paper, the inefficiency in the existing information-based method of the compressed measurement matrix design is solved. In particular, in contrast to the iterative optimizations of the measurement matrices in the literature, the DNNs are trained once based on the TSI optimization and are repeatedly implemented to detect the FHSS signals in an efficient manner. Thus, the practical online adaptivity of the FHSS measurement and detection can be achieved with the method proposed in this paper. From the signal processing aspect, the adaptivity in the FHSS signal processing based on the DNNs is also achieved.

The remainder of this paper is organized as follows: In Section 2, the problem formulation, including the signal and compressed measurement and detection models, are first rendered. In Section 3, the principles of the energy detection and the adaptive measurement kernel design based on the TSI optimization are described. Then in Section 4, the proposed adaptive measurement and detection method of the FHSS signals based on the fusion of the posterior information optimization and the DNNs is detailed. In Section 5, we provide the simulation results to verify the proposed method. Finally, in Section 6, the conclusions are drawn. It is worth mentioning that although the analysis and simulations were performed with the assumptions that single FHSS signal is present in the frequency band of interest, the proposed method can also be used for the multiple FHSS signal case.

## 2. Problem Formulation

In this paper, we propose a method to measure and detect the FHSS signals compressively, adaptively and efficiently. The proposed method was verified through simulations, where the Gauss binary frequency shift keying FHSS signal of the Bluetooth standard [50] were used, as a representative of the FHSS signals. Within each hopping period, the expression of the Bluetooth signal is given as follows:

(1) $$s\left(t\right)=\sqrt{\frac{E_{s}}{T_{s}}}exp\left[2\pi jf_{c}t+2\pi jh\sum_{r=1}^{N_{s}}c_{r}g\left(t-rT_{s}\right)\right],$$

where $T_{s}$ and $E_{s}$ represent the symbol period and the energy of the signal in a symbol period, respectively. $N_{s}$ represents the number of symbol periods in a hopping period. *h* is the modulation index of the FHSS signal. $c_{r}\in \left\{-1,1\right\}$ ($1\;\leq \;r\;\leq \;N_{s}$) is the *r*th symbol content in the hopping period. The function g(t) is the Gauss filtering item and can be expressed as:

(2) $$g\left(t\right)=\frac{Q(\alpha \left(t-\frac{T_{s}}{2}\right))-Q(\alpha \left(t+\frac{T_{s}}{2}\right))}{2T_{s}},$$

where $Q\left(x\right)=\int_{x}^{\infty }\frac{exp(-\frac{x^{2}}{2})}{\sqrt{2\pi }}$ is the Q-function and $\alpha =\frac{\pi }{T_{s}\sqrt{log(2)}}$.

As specified in the Bluetooth standard, the carrier frequency of the signal randomly hops among 79 channels from 2.402 to 2.480 GHz, with 1 MHz bandwidth for each channel. The symbol period takes the value of 1 μs and the frequency-hopping period is 625 μs, i.e., 625 symbol periods.

**Figure 1:**
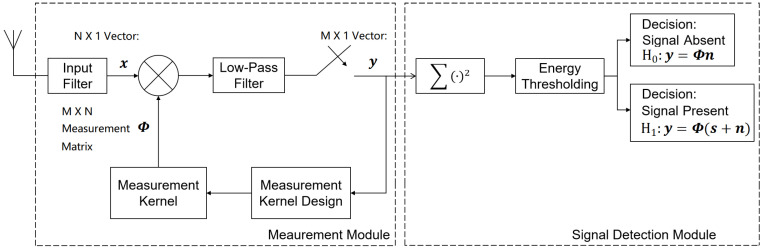
The adaptive FHSS compressed measurement and detection framework.

In this paper, the proposed adaptive measurements and detection of the FHSS signals are done compressively and non-cooperatively using the framework described in Figure 1.

In Figure 1, the wireless channel output is first passed through an input band-pass filter to remove the frequency components that are out of the range of interest. The output from the band-pass filter is then multiplied with the measurement kernels, and then integrated using a low-pass filter. The result from the low-pass filter is sampled at a compressed sampling rate, compared to the Nyquist rate respective to the entire FHSS hopping range. The measurement results are collected, and the measurement energy is calculated. Finally, the resulting energy is used to determine if the signal is present. The measurement kernels are designed sequentially and adaptively based on posterior knowledge of the channel output in a row-by-row manner, given the existing measurement data. Therefore, feedback is added from the measurement data to the measurement kernel design module.

The compressed measurement using the framework described in Figure 1 can be modeled as:

(3) y=Φx,

where the N×1 vector x represents the Nyquist sampled result from the input low-pass filter, regarding to the entire FHSS hopping range. The M×1 (M<<N) vector y represents the vector of the measurement data. Let us denote the FHSS frequency-hopping range as *B*. Then, using the framework in Figure 1, the M×N measurement matrix Φ is a block diagonal matrix with each block as an 1×CR vector, where

(4) $$CR=\frac{N}{M}=\frac{BT_{s}}{M}$$

is defined as the compression ratio (CR) in the compressed measurement. With the system framework described in Figure 1, each row block in the measurement matrix is defined as the measurement kernel of a single measurement. In this work, we normalize each row of Φ to unit energy before using them in the measurements. Thus, the rows of Φ are orthonormal to each other.

Based on the compressed measurements, the detection decision is made from the two following hypotheses:

(5) $$\begin{array}{cc} \; & H_{0}:y=\Phi n\\ \; & H_{1}:y=\Phi \left(s+n\right), \end{array}$$

where $H_{0}$ and $H_{1}$ denote the signal absent and signal present hypotheses, respectively. n denotes the channel noise, which is modeled as complex Gaussian white noise. The noise variance is denoted by $\sigma_{n}^{2}$ in this paper. s denotes the Nyquist rate sampled FHSS signal of s(t) in Equation (1) regarding to the entire FHSS hopping range.

In this paper, the signal detection is conducted based on the energy of the measurement data, and the measurement matrix is designed adaptively based on the gradually obtained measurement data. The principles of the proposed methods are detailed in the following sections.

## 3. The Theory of the Adaptive FHSS Signal Compressed Measurement and Detection

According to the noise folding theory [51], as the rows of the measurement matrix for Figure 1 are orthonormal to each other, the noise components in the measurement data are identically and independently distributed (i.i.d.) zero-mean complex Gaussian components with the variance $\sigma_{n}^{2}$. Then, the probability density function (PDF) of the measurement energy in the signal absent case can be modeled as follows:

(6) $$p_{r}\left(\lambda |H_{0}\right)=\frac{\lambda^{M-1}e^{-\frac{\lambda }{\sigma_{n}^{2}}}}{\sigma_{n}^{2M}M!},$$

where λ denotes the energy of the measurement data, and M! represents the factorial operation of the number of compressed measurements *M*.

The signal detection is conducted by energy thresholding. More specifically, given a positive threshold *T*, the theoretical false positive rate (FPR, i.e., false-alarm rate) is:

(7) $$FPR=\int_{T}^{\infty }p_{r}\left(\lambda |H_{0}\right)d\lambda$$

The measurement kernels in Figure 1 is adaptively and sequentially designed for each single measurement at a time, based on the measurements that have been already obtained and the TSI optimization. In this paper, we assume that no frequency hops happen during a FHSS detection process. Then, we define the TSI in the signal-detection task as the mutual information between the pre-filtered channel output and the measurement result, conditional on the existing measurement kernels and data. With the kernel of the first measurement randomly initialized, the measurement kernel in the *k*th (2≤k≤M) measurement is designed by solving the following problem:

(8) $$\begin{array}{cc} \; & \Phi_{k}=\max_{{\hat{\Phi }}_{k}}I\left(x_{k};y_{k}|\Lambda_{k-1},{\hat{\Phi }}_{k}\right),\\ \; & s.t.\;y_{k}={\hat{\Phi }}_{k}x_{k}\;\mathrm{and}\;{∥{\hat{\Phi }}_{k}∥}_{l_{2}}=1 \end{array}$$

In Equation (8), $\Lambda_{k-1}=\left\{\Phi_{1},\Phi_{2},\ldots ,\Phi_{k-1},y_{1},y_{2},\ldots ,y_{k-1}\right\}$, where $\Phi_{v}$ and $y_{v}$ (1≤v<k) represent the measurement kernel and the measurement result during the *v*th measurement, respectively. $\Phi_{k}$, $x_{k}$ and $y_{k}$ represent the measurement kernel, pre-filtered channel output and the measurement result, which are to be designed, observed and obtained at the *k*th measurement, respectively. ${∥\cdot ∥}_{l_{2}}$ represents the l−2 norm operation.

According to the information theory,

(9) $$I\left(x_{k};y_{k}|\Lambda_{k-1},{\hat{\Phi }}_{k}\right)=h\left(y_{k}|\Lambda_{k-1},{\hat{\Phi }}_{k}\right)-h\left(y_{k}|x_{k},\Lambda_{k-1},{\hat{\Phi }}_{k}\right),$$

where h(·|·) denotes the conditional entropy. To simplify, we further assume that the measurements made at different times are independent of each other. Then, $h\left(y_{k}|x_{k},\Lambda_{k-1},{\hat{\Phi }}_{k}\right)=h\left(y_{k}|x_{k},{\hat{\Phi }}_{k}\right)$ only depends on the variance of the channel noise, and thus is a constant. Therefore, the optimization problem in Equation (8) is equivalent to:

(10) $$\begin{array}{cc} \; & \Phi_{k}=\max_{{\hat{\Phi }}_{k}}h\left(y_{k}|\Lambda_{k-1},{\hat{\Phi }}_{k}\right),\\ \; & s.t.\;y_{k}={\hat{\Phi }}_{k}x_{k}\;\mathrm{and}\;{∥{\hat{\Phi }}_{k}∥}_{l_{2}}=1 \end{array}$$

To solve the optimization problem in Equation (10), we model the pre-filtered channel output during the *k*th compressed measurement, i.e., $x_{k}$, using the mixture of Gaussian (moG) models, which were usually implemented to solve signal processing problems with information analysis and optimizations in the literature [39,52,53,54]. In this paper, we uniformly divide the entire FHSS frequency-hopping range into *L* subbands and model the posterior distribution of $x_{k}$ as:

(11) $$p_{r}\left(x_{k}|\Lambda_{k-1}\right)=P_{r}\left(H_{0}|\Lambda_{k-1}\right)f_{0}\left(x_{k}\right)+P_{r}\left(H_{1}|\Lambda_{k-1}\right)\sum_{l=1}^{L}P_{r}\left(B_{l}|H_{1},\Lambda_{k-1}\right)f_{l}\left(x_{k}\right),$$

where $P_{r}\left(H_{0}|\Lambda_{k-1}\right)$, $P_{r}\left(H_{1}|\Lambda_{k-1}\right)$ and $P_{r}\left(B_{l}|H_{1},\Lambda_{k-1}\right)$ (1≤l≤L) are the probabilities of signal absent, signal present cases and the probability that the *l*th subband is occupied in the signal present case, respectively, given the measurement kernels and data from the 1st through the (k−1)th measurements. The item $f_{0}\left(x_{k}\right)=CN\left(0,\;C_{\mathrm{nn}}\right)$ represents the zero-mean complex Gaussian component of the signal absent case, where $C_{\mathrm{nn}}$ is the diagonal covariance matrix with the diagonal entries as $\sigma_{n}^{2}$. $f_{l}\left(x_{k}\right)=CN\left(0,\;C_{\mathrm{xx},l}\right)$ represent the Gaussian component where the *l*th subband is occupied in the signal present case. The covariance matrix in this case can be expressed as:

(12) $$C_{\mathrm{xx},l}=C_{\mathrm{ss},l}+C_{\mathrm{nn}},$$

where $C_{\mathrm{ss},l}$ represents the covariance matrix of the noise-free FHSS signal falling in the *l*th subband, modeled as complex Gaussian white noise within the *l*th subband.

With the signal model in Equation (11), it can be proved that the item for the signal absent case does not affect the result of the measurement optimization problem, and Equation (10) is equivalent to:

(13) $$\begin{array}{cc} \; & \Phi_{k}=\max_{{\hat{\Phi }}_{k}}h\left(y_{k}|H_{1},\Lambda_{k-1},{\hat{\Phi }}_{k}\right),\\ \; & s.t.\;y_{k}={\hat{\Phi }}_{k}x_{k}\;\mathrm{and}\;{∥{\hat{\Phi }}_{k}∥}_{l_{2}}=1, \end{array}$$

where

(14) $$h\left(y_{k}|H_{1},\Lambda_{k-1},{\hat{\Phi }}_{k}\right)\approx -log\left[\sum_{l=1}^{L}\frac{P_{r}\left(B_{l}|H_{1},\Lambda_{k-1},{\hat{\Phi }}_{k}\right)}{\pi ({\hat{\Phi }}_{k}C_{\mathrm{xx},l})}\right]$$

The posterior probabilities of the subband usages given the signal present hypothesis, i.e., $P_{r}\left(B_{l}|H_{1},\Lambda_{k-1}\right)$ (1≤l≤L) in Equation (11), are updated as the adaptive measurements proceeds, based on the Bayes rule. With the measurement results modeled as independent to each other, the Bayes update can be expressed as:

(15) $$\begin{array}{cc} P_{r} & \left(B_{l}|H_{1},\Lambda_{k-1}\right)=P_{r}\left(B_{l}|H_{1},\Lambda_{k-2},\Phi_{k-1},y_{k-1}\right)\\ \; & =\frac{p_{r}\left(B_{l},y_{k-1}|H_{1},\Lambda_{k-2},\Phi_{k-1}\right)}{p_{r}\left(y_{k-1}|H_{1},\Lambda_{k-2},\Phi_{k-1}\right)}\\ \; & =\frac{P_{r}\left(B_{l}|H_{1},\Lambda_{k-2},\Phi_{k-1}\right)p_{r}\left(y_{k-1}|H_{1},B_{l},\Lambda_{k-2},\Phi_{k-1}\right)}{\sum_{l=1}^{L}P_{r}\left(B_{l}|H_{1},\Lambda_{k-2},\Phi_{k-1}\right)p_{r}\left(y_{k-1}|H_{1},B_{l},\Phi_{k-2},\Phi_{k-1}\right)}\\ \; & =\frac{P_{r}\left(B_{l}|H_{1},\Lambda_{k-2}\right)p_{r}\left(y_{k-1}|B_{l},\Phi_{k-1}\right)}{\sum_{l=1}^{L}P_{r}\left(B_{l}|H_{1},\Lambda_{k-2}\right)p_{r}\left(y_{k-1}|B_{l},\Phi_{k-1}\right)}, \end{array}$$

where $P_{r}\left(B_{l}|H_{1},\Lambda_{0}\right)=P_{r}\left(B_{l}|H_{1}\right)$ represents the prior probability that the *l*th subband is used in the signal present case. The likelihood function $p_{r}\left(y_{k-1}|B_{l},\Phi_{k-1}\right)$ is given by:

(16) $$p_{r}\left(y_{k-1}|H_{1},B_{l},\Phi_{k-1}\right)=\frac{1}{\pi \Phi_{k-1}C_{\mathrm{xx},l}\Phi_{k-1}^{H}}e^{-\frac{|y_{m-1}{|}^{2}}{\Phi_{k-1}C_{\mathrm{xx},l}\Phi_{k-1}^{H}}},$$

where ${\left(\cdot \right)}^{H}$ represents the Hermitian operation.

In the literature, the Shannon information-based optimization problems are usually solved with recursive gradient methods [41,50,51]. For the optimization problem in Equation (13), an update step for the measurement kernel in the recursive optimization process can be expressed as:

(17) $$\begin{array}{cc} \; & {\tilde{\Phi }}_{k}^{\left(u+1\right)}=\Phi_{k}^{\left(u\right)}+\mu \nabla_{\Phi_{k}}h\left(y_{k}|H_{1},\Lambda_{k-1},\Phi_{k}^{\left(u\right)}\right),\\ \; & \Phi_{k}^{\left(u+1\right)}=\frac{{\tilde{\Phi }}_{k}^{\left(u+1\right)}}{∥{\tilde{\Phi }}_{k}^{\left(u+1\right)}∥}, \end{array}$$

where $\Phi_{k}^{\left(u\right)}$ and $\Phi_{k}^{\left(u+1\right)}$ represents the resulting measurement kernel at the *k*th row of the measurement matrix at the *u*th and ${\left(u+1\right)}^{t}h$ iterations, respectively. μ is the optimization step. According to Equation (14), the gradient can be found by:

(18) $$\nabla_{\Phi_{k}}h\left(y_{k}|H_{1},\Lambda_{k-1},\Phi_{k}\right)\approx \frac{\sum_{l=1}^{L}P_{r}\left(B_{l}|H_{1},\Lambda_{k-1}\right){\left(\Phi_{k}C_{\mathrm{xx},l}\Phi_{k}^{H}\right)}^{-2}\Phi_{k}C_{\mathrm{xx},l}^{H}}{\sum_{l=1}^{L}P_{r}\left(B_{l}|H_{1},\Lambda_{k-1}\right){\left(\Phi_{k}C_{\mathrm{xx},l}\Phi_{k}^{H}\right)}^{-1}}$$

For interested readers, the derivations to Equations (13), (14) and (18) are provided in Appendix A.

## 4. Knowledge-Enhanced Compressed Detection of Frequency-Hopping Spread Spectrum Signals with Deep Neural Networks

Theoretically, the signal detection with a measurement matrix from the recursive optimization in Equation (17) can acquire improved detection accuracy, compared to the compressed detection with random measurement kernels. However, according to simulations, it usually needs more than 20,000 iterations to converge the optimization process and result in significantly improved detection performance, which usually leads to a time-consuming process. Therefore, it is not feasible for online adaptive measurement kernel design and signal-detection implementations. To improve the efficiency of the method, we propose an DNN-based method to conduct the adaptive measurements and detection of the FHSS signals. In contrast to the recursive method described in Equation (17), the neural networks in the proposed method are trained once and used for adaptive measurement kernel design, repeatedly.

### 4.1. The Structure and the Training of the Deep Neural Networks

**Figure 2:**
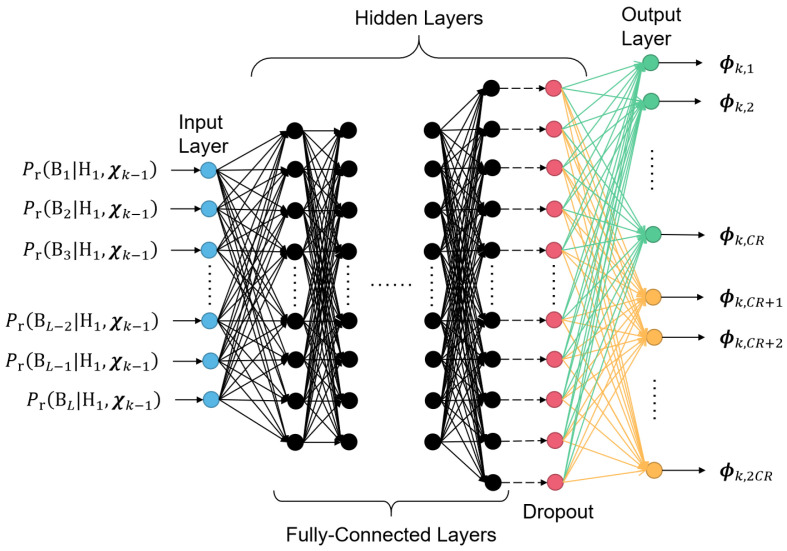
The architecture of the deep neural networks used in the adaptive design of the measurement kernels.

The structure of the DNNs in the proposed method is shown in Figure 2.

As described in Figure 2, the architecture of the proposed DNNs is fully connected. The nodes in the input layer represent posterior probabilities of the subband usage given the designed coefficients in the measurement matrix and the measured results in the signal present case, i.e., $P_{r}\left(B_{l}|H_{1},\Lambda_{k-1}\right)$ (1≤l≤L) in Equation (15). The width of the input layer is equal to the number of subbands divided in the moG model of the FHSS signals. The output layer of the DNN contains the CR designed coefficients in the measurement kernel of a measurement. Considering that signals and the coefficients of the measurement kernels are in the complex form, the width of output layer is 2CR, where CR nodes represent the real part, and the others represent the imaginary parts. If the depth of neural network is too high, the training process becomes difficult to converge; while if the depth of the neural network is too low, the resulting measurement matrix may not be effective enough. With simulation trials on various structures of the DNNs, we find that the 8-layer deep neural networks are efficient to train, and meanwhile effectively improve the accuracy in the adaptive FHSS signal detection. For the six hidden layers in the proposed DNN, the width of 1st through the 5th hidden layers is 512 and the width of the 6th hidden layer is 1024. With the nodes in the input layer denoted with the row vector $q_{0}$, the nodes in the *m*th (1≤m≤6) hidden layers are then calculated by:

(19) $$q_{m}=\tanh\left(q_{m-1}\cdot W_{m}\right),$$

where $q_{m}$ and $q_{m-1}$ (m≤1) are row vectors, representing the nodes in the *m*th and (m−1)th layers of the DNN, respectively. $W_{m}$ is the matrix of weights in the matrix multiplication to obtain nodes of the *m*th layer. tanh(·) is the entry-wise hyperbolic tangent activation function, which can be expressed as:

(20) $$tanh\left(z\right)=\frac{\sinh(z)}{\cosh(z)}=\frac{e^{z}-e^{-z}}{e^{z}+e^{-z}}$$

In our study, we found that deterministic input-output relationships in the feedback route could lead to a wrong convergence for the adaptive measurement and decision procedure of the entire system, if there exists even a little ideality during the training of the DNN. To solve this problem, we add a dropout layer after the 6th hidden layer, with a dropping rate of 0.95. The dropout layer works in both the training process of the DNN and the adaptive FHSS measurement processing afterwards. Finally, a full-connection operation is added to generate the output layer. Therefore, we have:

(21) $$q_{out}=Dropout\left(q_{6},0.95\right)\cdot W_{\mathrm{out}},$$

where Dropout(·,0.95) represents the dropout operation that replaces 95% of the entries in the vector with zeros. $W_{m}$ is the matrix of weights in the matrix multiplication to obtain nodes of the output layer. $q_{out}$ represents the nodes in the output layer.

In this work, the training of the DNNs was conducted using the gradient-based back-propagation method. The posterior subband usage probabilities from the simulations of the adaptive measurement and detection process using the conventional recursive optimization method were collected and used as the training data. The negativity of the conditional differential entropy in Equation (13), $-h(y_{k}|H_{1},\Lambda_{k-1},\Phi_{k})$, was used for the training penalty, which could be approximated as a function of the data to input layer and the result from output layer, i.e., $P_{r}\left(B_{l}|H_{1},\Lambda_{k-1}\right)$ and $\Phi_{k}$, according to Equation (14). The number of subbands in Equation (14) was taken to be L=20.

The DNN training was conducted using the TensorFlow 2.0 GPU version [55] based on Python 3.7 on a computer with the NVDIA Quadro P2000 GPU. Each of the proposed DNNs was trained using 20,000 training samples with the batch size of 100 and 400 training epochs in total. To ensure that the noise components in the measurement results were i.i.d. Gaussian components, the designed coefficients from the DNNs were further normalized to unit l−2 norm before used in the measurement.

### 4.2. Combination of Knowledge-Enhanced Compressed Detection Architecture and the Deep Neural Networks

**Figure 3:**
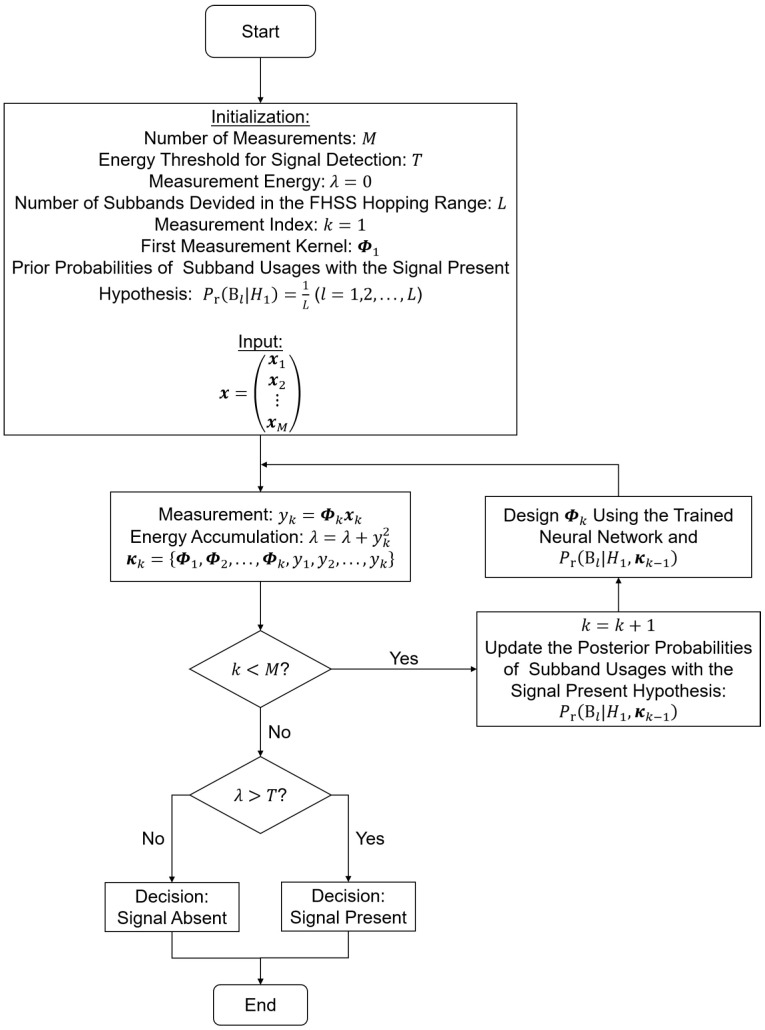
The Proposed Procedure of the Adaptive Compressed Measurement and Detection of the FHSS Signals.

With the trained DNNs, the proposed procedure of the adaptive compressed measurement and detection of the FHSS signals can be described in Figure 3.

In the adaptive procedure described in Figure 3, the coefficients of the measurement kernel in the first measurement are initialized using complex Gaussian identically independent distributions, and then normalized to unit l−2 norm. The prior probabilities of the subband usage in the signal present case, i.e., $P_{r}\left(B_{l}|H_{1}\right)$ (1≤l≤L) are taken to be equal to each other, as no prior knowledge of the subband usage is assumed.

The first measurement result is obtained using the initialized measurement kernel. Then the posterior probabilities of the subband usages are calculated according to Equation (15), and passed to the trained neural network to design the measurement kernel for the next measurement. In this manner, the measurements, the subband usage posterior probability updates and the measurement kernel design steps with the DNN are done sequentially and iteratively until the entire measurement procedure is finished. Finally, the energy of the measurement data, i.e., the resulting λ in Figure 3, is used to make the detection decision. As described in Section 3, if measurement energy is smaller than the decision threshold *T*, the signal absent decision is made; otherwise, the signal present decision is made.

## 5. Results

In this section, we provide Monte-Carlo simulation results to evaluate the performance of the proposed method. We first studied the detection accuracy performance of the proposed adaptive compressed detection method. In comparison, the non-compressed detection and the conventional compressed detection methods using the system in Figure 1 were simulated. In the case of conventional compressed detection method, the measurement kernels were selected according to the identical independent complex Gaussian distributions, and then each row of the measurement matrix was normalized to unit energy. In addition, the FHSS signal compressed detection method [42] rendered in 2020, which was conducted based on partial DFT and maximum energy thresholding of the compressed samples, with each measurement kernel taking up an entire row of the measurement matrix, was also studied in comparison. To obtain fair comparisons of the signal-detection performances for different methods, we took energy thresholds *T* for the proposed method, the non-compressed method and the conventional compressed method according to Equations (6) and (7) by taking the theoretical FPR as 0.01 (In fact, any FPR value between 0 and 1 is valid). The threshold for the partial DFT-based method at each CR and each SNR was determined by taking the simulated FPR as 0.01 from 10,000 simulations.

**Figure 4:**
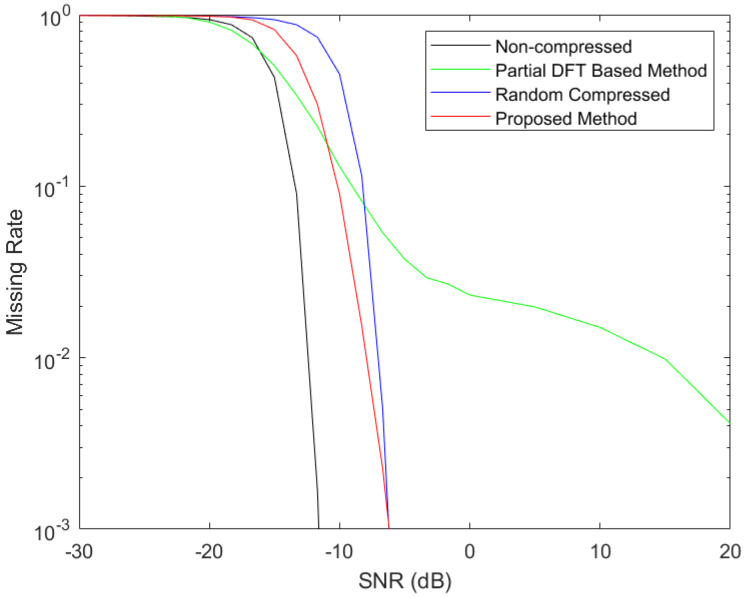
FHSS Signal-Detection Performance at CR=10.

**Figure 5:**
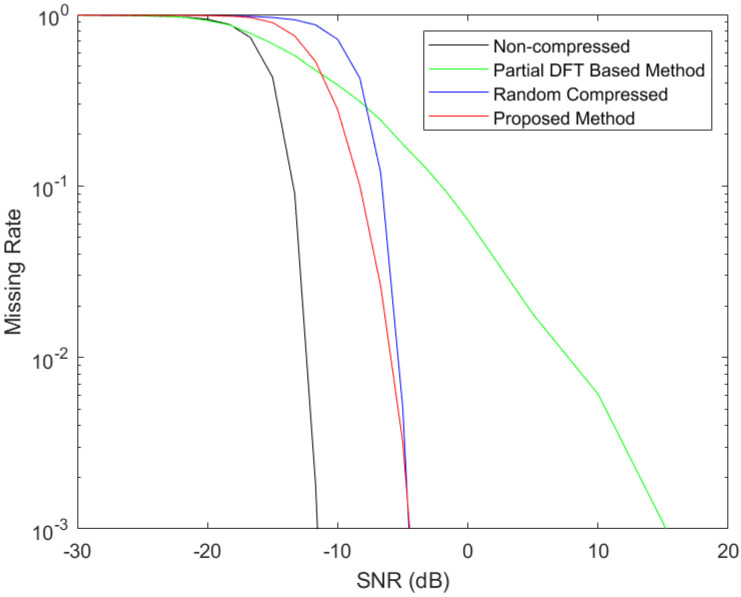
FHSS Signal-Detection Performance at CR=20.

The simulated curves of missing detection rates versus the signal-to-noise ratio (SNR) are shown in Figure 4 and Figure 5, where the CRs were taken to be 10 and 20, respectively. The DNNs to design the measurement kernels were trained using the TensorFlow 2.0 GPU version based on Python 3.7 on a computer with the NVIDIA Quadro P2000 GPU. To train the DNN for the adaptive measurement kernel design at *CR* = 10, it took 5.36 days. To train the DNN for the adaptive measurement kernel design at *CR* = 20, it took 6.61 days. The SNR is defined as the ratio between the signal power and the noise power. As specified above, the number of Nyquist samples during the measurement procedure were N=6400, resulting in M=640 and M=320 for the two CR cases in the compressed measurement methods. To generate the curves in Figure 4 and Figure 5, the SNR varied from −30 dB to 20 dB. Each point in the curves was generated using 100,000 simulations. The FHSS carrier frequency at each simulation was randomly selected from the 79 channels with equal probabilities.

From the two figures, we observe that at any CR and certain false-alarm rate (i.e., false positive rate), the missing rates (i.e., false negative rate) from all the four methods in comparison decrease with increased SNRs. Non-compressed detection achieves the lowest missing rate for most cases. The partial DFT-based compressed detection method can obtain good detection performance at median SNR values. However, for higher SNR cases, the missing detection rates can be even significantly higher than the conventional compressed detection with random measurement kernels. The adaptive compressed detection method, although obtaining higher missing rates than the non-compressed detection method and obtaining higher missing rates than the partial DFT-based compressed detection method at some median SNR values, outperforms the compressed method with random measurement kernels for low and median SNR values at any CR. This improvement in terms of missing rate can even be around an order at some SNRs. For high SNR values (above −6.5 dB at CR=10 and above −5 dB at CR=20), the missing detection rates of the proposed method fall below 0.001, are close to those of the conventional compressed detection method with random measurement kernels.

**Figure 6:**
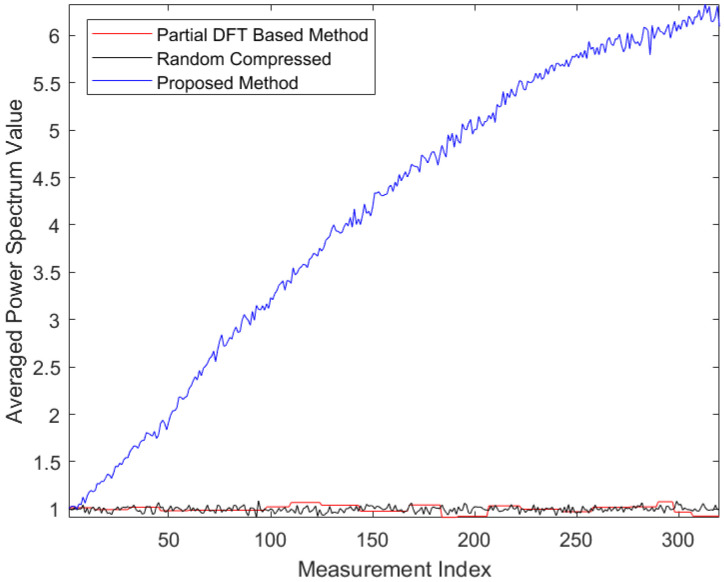
Evolution of the Averaged Power Spectrum Value at the True Subbands at CR=20 and SNR=−10 dB.

To have a deeper insight into the procedure of the adaptive measurements in the proposed method, we performed a further analysis on the power spectra of the designed measurement kernels. A larger value of the measurement kernel power spectrum value within the true subband that the FHSS signal falls in indicates a higher SNR resulted in the measurement data. In turn, higher detection accuracy would be expected. As a representative, Figure 6 shows the averaged power spectrum value of the adaptive measurement kernels on the true FHSS subbands versus the measurement index at CR=20 and SNR=−10 dB over 100,000 adaptive compressed detection simulations. In each of the simulations, the carrier frequency was randomly selected from the 79 channels. In comparison, the averaged power spectrum value of the measurement kernels on the true subbands in the partial DFT-based method and that of the conventional random measurement kernels versus the measurement index at CR=20 over 100,000 compressed detection simulations are also plotted in Figure 6.

In Figure 6, we observe that the averaged power spectrum value of the adaptive measurement kernels within the true subbands that the FHSS signals fall in increases gradually as more measurements are done. In this case, a gradual reduction of the subband usage uncertainty is obtained. In contrast, there are no incremental trends of averaged power spectrum value for the random measurement kernels and the measurement kernels from partial DFT-based method during the measurement process, as those measurement kernels are designed prior to the measurement processes.

In addition to the simulations on the detection accuracy and the measurement kernel components above, we also conducted simulations to compare the time costs of the proposed adaptive detection method using the DNNs and that using recursive measurement kernel optimization method discussed in Section 2. In addition, the time cost partial DFT-based compressed detection method in [42] was also compared. The cases at CR=10 and CR=20 were both studied, and 100 FHSS detection simulations at SNR=−10 dB were done for each of the methods at each CR case. To achieve the detection accuracy of the proposed method at such CR values, 20,000 iterations are usually needed for the recursive method to design the measurement kernel of a single measurement, which was implemented in the simulations. Similar to the theoretical analysis and simulation discussed above, 6400 Nyquist samples respective to the entire FHSS hopping range were included to decide whether the signal is present or absent for each simulation, resulting in 640 and 320 measurements in each detection simulation for CR=10 and CR=20, respectively. A computer with the CPU of Intel Xeon E3-1225 v5 @ 3.30 GHz and the RAM size of 16.0 GB was implemented to run these simulations. The statistics of the timing results to conduct each of these 100 detection simulations are shown in Table 1.

From Table 1, we observe that as the measurement matrix for the partial DFT-based method is designed non-adaptively prior to the measurement process, the partial DFT-based method cost the least time in the measurement and detection process. More importantly, from the timing results of the two adaptive methods, we observed that the efficiency of the proposed adaptive method is significantly improved, compared to the method with recursively optimized measurement kernels. In the case of CR=10, this improvement can be more than 320 times on average; while in the case of CR=20, the efficiency improvement can reach more than 380 times on average. Although the time costs of the proposed method as stated in Table 1 still seems relatively long for the practical FHSS detections, the implementation efficiency can be expected to be further improved considerably with specifically designed hardware and software modules to implement the DNN and the signal detection in this paper. The designing of such modules will be studied in our future work.

## 6. Conclusions

In this paper, a knowledge-enhanced compressed measurement method was proposed for adaptive and non-cooperative detection of the FHSS signals using the DNNs. In contrast to the conventional non-compressed receiver, which was unfeasible for wide frequency-hopping bandwidths, the proposed method in this paper conducted the FHSS signal detection with compressed sampling rates. The measurement kernels were designed adaptively based on the continuously updated knowledge from the compressed measurement. Moreover, in contrast to the iterative measurement kernel optimizations, the DNNs was trained once off-line based on the TSI optimization, and implemented repeatedly online to adaptively design the measurement kernels, enabling efficient FHSS signal detection. Simulation results showed that the proposed adaptive compressed detection method achieved stably low missing detection rates, compared to the compressed detection system with random measurement kernels and the recently proposed work. In addition, through the simulations, we also showed that the efficiency of the proposed adaptive FHSS detection method with the implementation of the DNNs was proved to be significantly higher than that using the recursive measurement kernel optimization methods. Thus, the measurement kernel design procedure improved its efficiency significantly, and became much more practical for the online adaptive measurements and detection of FHSS signals.

## Figures and Tables

**Table 1:** Comparison of Time Costs between the Proposed Adaptive Compressed FHSS Measurement and Adaptive Compressed FHSS Measurement with Recursive Measurement Kernel Optimization for Each of the 100 Detection Simulations.

Compression Ratio	Method	Maximum Time Cost	Minimum Time Cost	Average Time Cost
10	Partial DFT-Based Method	0.0217 s	0.0052 s	0.0098 s
	Proposed Adaptive Method	14.1356 s	5.3613 s	10.5885 s
	Recursively Optimized Adaptive Method	4221.7262 s	2674.4538 s	3492.5201 s
20	Partial DFT-Based Method	0.0133 s	0.0025 s	0.0049 s
	Proposed Adaptive Method	6.5995 s	2.5448 s	5.2072 s
	Recursively Optimized Adaptive Method	2509.2800 s	1526.8290 s	1991.2884 s

## Data Availability

Not applicable.

## Abbreviations

The following abbreviations are used in this manuscript:

SS	Spread spectrum
FHSS	Frequency-hopping spread spectrum
CS	compressed sensing
DFT	discrete Fourier transform
ANN	Artificial neural network
GPU	Graphic processing unit
DNN	Deep neural network
TSI	Task-specific information
CR	Compression ratio
i.i.d.	Identically and independently distributed
PDF	Probability density function
FPR	False positive rate
moG	Mixture of Gaussian
SNR	Signal-to-noise ratio

## Appendix A. Derivations on Equations (13), (14) and (18)

In the Appendix of this paper, we provide the detailed derivations of Equations (13), (14) and (18) in Section 3.

In the measurement kernel design stage, the pre-filtered signal from the wireless channel is modeled with an moG distribution as described in Equation (11). As a compressed measurement can be treated as a weighted sum of neighbored Nyquist samples, the distribution of the measured result $y_{k}$ (1≤k≤M), given the measurement kernel $\Phi_{k}$ and the measurement kernels and data in the 1st through the *k*th measurements is also an moG distribution:

(A1) $$p_{r}\left(y_{k}|\Lambda_{k-1},\Phi_{k}\right)=P_{r}\left(H_{0}|\Lambda_{k-1}\right)g_{0}\left(y_{k}|\Phi_{k}\right)+\sum_{l=1}^{L}P_{r}\left(H_{1}|\Lambda_{k-1}\right)P_{r}\left(B_{l}|H_{1},\Lambda_{k-1}\right)g_{l}\left(y_{k}|\Phi_{k}\right),$$

where $P_{r}\left(H_{0}|\Lambda_{k-1}\right)$, $P_{r}\left(H_{1}|\Lambda_{k-1}\right)$ and $P_{r}\left(B_{l}|H_{1},\Lambda_{k-1}\right)$ represent the posterior probabilities of the signal absent case, the signal present case and the probability that the *l*th subband is occupied in the signal present case, respectively, given the measurement kernels and data during the 1st through the (k−1)th measurements (i.e., $\Lambda_{k-1}$). $g_{0}\left(y_{k}|\Phi_{k}\right)$ and $g_{l}\left(y_{k}|\Phi_{k}\right)$ (1≤l≤L) denote the Gaussian component in the signal absent case and the Gaussian component where the *l*th subband is occupied in the signal present case, respectively. Let CN(·,·) stand for the PDF of the complex Gaussian distribution with the first and second parameters representing the mean and covariance matrix/variance for the distribution, respectively. As the rows of the measurement matrix are normalized to unit energy, we have:

(A2) $$g_{0}\left(y_{k}|\Phi_{k}\right)=CN\left(0,\Phi_{k}C_{\mathrm{nn}}\Phi_{k}^{H}\right)$$

and

(A3) $$g_{l}\left(y_{k}|\Phi_{k}\right)=CN\left(0,\Phi_{k}C_{\mathrm{xx}}^{\left(l\right)}\Phi_{k}^{H}\right),$$

where $C_{\mathrm{nn}}$ is diagonal matrix with its diagonal entries equal to the channel noise variance $\sigma_{n}^{2}$. $C_{\mathrm{xx}}^{\left(l\right)}=C_{\mathrm{ss}}^{\left(l\right)}+C_{\mathrm{nn}}$, with $C_{\mathrm{ss}}^{\left(l\right)}$ representing the covariance matrix of the noise-free FHSS signal that falls into the *l*th subband in the signal present case.

According to the information theory, the differential entropy of the measurement result $y_{k}$ given the measurement kernels and data in the 1st through the (k−1)th measurements and the measurement kernel in the *k*th measurement can be expressed as:

(A4) $$\begin{array}{cc} \; & h(y_{k}|\Lambda_{k-1},\Phi_{k})\\ = & -\int p_{r}\left(y_{k}|\Lambda_{k-1},\Phi_{k}\right)log[p_{r}\left(y_{k}|\Lambda_{k-1},\Phi_{k}\right)]dy_{k}\\ = & -\int p_{r}\left(y_{k}|\Lambda_{k-1},\Phi_{k}\right)log[P_{r}\left(H_{0}|\Lambda_{k-1}\right)g_{0}\left(y_{k}|\Phi_{k}\right)\\ \; & +\sum_{l=1}^{L}P_{r}\left(H_{1}|\Lambda_{k-1}\right)P_{r}\left(B_{l}|H_{1},\Lambda_{k-1}\right)g_{l}\left(y_{k}|\Phi_{k}\right)]dy_{k} \end{array}$$

Using Taylor expansion at $y_{k}=0$, the logarithm item in Equation (A4) can be expressed as:

(A5) $$\begin{array}{cc} \; & log\left[P_{r}\left(H_{0}|\Lambda_{k-1}\right)g_{0}\left(y_{k}|\Phi_{k}\right)+\sum_{l=1}^{L}P_{r}\left(H_{1}|\Lambda_{k-1}\right)P_{r}\left(B_{l}|H_{1},\Lambda_{k-1}\right)g_{l}\left(y_{k}|\Phi_{k}\right)\right]\\ = & log\left[P_{r}\left(H_{0}|\Lambda_{k-1}\right)g_{0}\left(0|\Phi_{k}\right)+\sum_{l=1}^{L}P_{r}\left(H_{1}|\Lambda_{k-1}\right)P_{r}\left(B_{l}|H_{1},\Lambda_{k-1}\right)g_{l}\left(0|\Phi_{k}\right)\right]+\Sigma \left(0\right)y_{k}+\ldots , \end{array}$$

where

(A6) $$\begin{array}{cc} \Sigma (0)= & \nabla y_{k}log\left[g(y_{k}|\Lambda_{k-1},\Phi_{k})\right]{|}_{y_{k}=0}\\ = & \nabla y_{k}log\left[P_{r}\left(H_{0}|\Lambda_{k-1}\right)g_{0}\left(y_{k}|\Phi_{k}\right)+\sum_{l=1}^{L}P_{r}\left(H_{1}|\Lambda_{k-1}\right)P_{r}\left(B_{l}|H_{1},\Lambda_{k-1}\right)g_{l}\left(y_{k}|\Phi_{k}\right)\right]{|}_{y_{k}=0} \end{array}$$

Approximating the logarithm in Equation (A4) with the first two items of its Taylor expansion in Equation (A5), Equation (A4) can be further derived as follows:

(A7) $$\begin{array}{cc} h & \left(y_{k}|\Lambda_{k-1},\Phi_{k}\right)\\ = & -\int p_{r}\left(y_{k}|\Lambda_{k-1},\Phi_{k}\right)[log[P_{r}\left(H_{0}|\Lambda_{k-1}\right)g_{0}\left(0|\Phi_{k}\right)\\ \; & +\sum_{l=1}^{L}P_{r}\left(H_{1}|\Lambda_{k-1}\right)P_{r}\left(B_{l}|H_{1},\Lambda_{k-1}\right)g_{l}\left(0|\Phi_{k}\right)]+\Sigma \left(0\right)y_{k}+\ldots ]dy_{k}\\ \approx & -log\left[P_{r}\left(H_{0}|\Lambda_{k-1}\right)g_{0}\left(0|\Phi_{k}\right)+\sum_{l=1}^{L}P_{r}\left(H_{1}|\Lambda_{k-1}\right)P_{r}\left(B_{l}|H_{1},\Lambda_{k-1}\right)g_{l}\left(0|\Phi_{k}\right)\right]\\ \; & \int p_{r}\left(y_{k}|\Lambda_{k-1},\Phi_{k}\right)dy_{k}-\Sigma \left(0\right)\int y_{k}p_{r}\left(y_{k}|\Lambda_{k-1},\Phi_{k}\right)dy_{k}\\ = & -log\left[P_{r}\left(H_{0}|\Lambda_{k-1}\right)g_{0}\left(0|\Phi_{k}\right)+\sum_{l=1}^{L}P_{r}\left(H_{1}|\Lambda_{k-1}\right)P_{r}\left(B_{l}|H_{1},\Lambda_{k-1}\right)g_{l}\left(0|\Phi_{k}\right)\right]\\ = & -log\left[\frac{P_{r}\left(H_{0}|\Lambda_{k-1}\right)}{\pi (\sigma_{n}^{2}\Phi_{k}\Phi_{k}^{H})}+P_{r}\left(H_{1}|\Lambda_{k-1}\right)\sum_{l=1}^{L}\frac{P_{r}\left(B_{l}|H_{1},\Lambda_{k-1}\right)}{\pi (\Phi_{k}C_{\mathrm{xx}}^{\left(k,l\right)}\Phi_{k}^{H})}\right]\;. \end{array}$$

As the posterior probability value of $P_{r}\left(H_{0}|\Lambda_{k-1}\right)$ becomes a constant in the optimization problem of the measurement kernel Φ, when the measurement kernels and data from the 1st to the (k−1)th measurements are known. Therefore, the optimization of Φ through the maximization of $h(y_{k}|\Lambda_{k-1},\Phi_{k})$ in Equation (10) is equivalent to the optimization problem described in Equation (13), with:

(A8) $$h\left(y_{k}|H_{1},\Lambda_{k-1},\Phi_{k}\right)\approx -log\left[\sum_{l=1}^{L}\frac{P_{r}\left(B_{l}|H_{1},\Lambda_{k-1}\right)}{\pi (\Phi_{k}C_{\mathrm{xx}}^{\left(k,l\right)}\Phi_{k}^{H})}\right]\;.$$

Using the chain rule of the gradient operation, we then obtain the following derivations from Equation (A8):

(A9) $$\begin{array}{cc} \; & \nabla_{\Phi_{k}}h\left(y_{k}|H_{1},\Phi_{k},\Lambda_{k-1}\right)\\ \; & \approx \nabla_{\Phi_{k}}\left\{-log\left[\sum_{l=1}^{L}\frac{P_{r}\left(B_{l}|H_{1},\Lambda_{k-1}\right)}{\pi (\Phi_{k}C_{\mathrm{xx}}^{\left(k,l\right)}\Phi_{k}^{H})}\right]\right\}\\ \; & =-\frac{\sum_{l=1}^{L}P_{r}\left(B_{l}|H_{1},\Lambda_{k-1}\right)\pi^{-1}\nabla_{\Phi_{k}}\left\{{\left(\Phi_{k}C_{\mathrm{xx}}^{\left(k,l\right)}\Phi_{k}^{H}\right)}^{-1}\right\}}{\sum_{l=1}^{L}P_{r}\left(B_{l}|H_{1},\Lambda_{k-1}\right)\pi^{-1}{\left(\Phi_{k}C_{\mathrm{xx}}^{\left(k,l\right)}\Phi_{k}^{H}\right)}^{-1}}\\ \; & =-\frac{\sum_{l=1}^{L}P_{r}\left(B_{l}|H_{1},\Lambda_{k-1}\right)\pi^{-1}{\left(\Phi_{k}C_{\mathrm{xx}}^{\left(k,l\right)}\Phi_{k}^{H}\right)}^{-2}\Phi_{k}{C_{\mathrm{xx}}^{\left(k,l\right)}}^{H}}{\sum_{l=1}^{L}P_{r}\left(B_{l}|H_{1},\Lambda_{k-1}\right)\pi^{-1}{\left(\Phi_{k}C_{\mathrm{xx}}^{\left(k,l\right)}\Phi_{k}^{H}\right)}^{-1}}\\ \; & =-\frac{\sum_{l=1}^{L}P_{r}\left(B_{l}|H_{1},\Lambda_{k-1}\right){\left(\Phi_{k}C_{\mathrm{xx}}^{\left(k,l\right)}\Phi_{k}^{H}\right)}^{-2}\Phi_{k}{C_{\mathrm{xx}}^{\left(k,l\right)}}^{H}}{\sum_{l=1}^{L}P_{r}\left(B_{l}|H_{1},\Lambda_{k-1}\right){\left(\Phi_{k}C_{\mathrm{xx}}^{\left(k,l\right)}\Phi_{k}^{H}\right)}^{-1}}\;. \end{array}$$

## References

[1] Zhu J. Spread spectrum techniques and applications in the Asia-Pacific region. Proceedings of the International Symposium on Spread Spectrum Techniques and Applications.

[2] Youness A., Naima K. (2019). A comprehensive survey on spectrum sensing in cognitive radio networks: Recent advances, new challenges, and future research directions. Sensors.

[3] Zheng J., Liao Z., Ma X., Jin Y., Ma H. (2022). Dense-frequency signal-detection based on the primal-dual splitting method. Entropy.

[4] Lv J., Qu W. Application of the wavelet rearrangement algorithm in the detection of noncooperative frequency hopping signals. Proceedings of the IEEE 11th International Conference on Signal Processing.

[5] Luo S., Luo L. Adaptive detection of an unknown FH signal based on image features. Proceedings of the 5th International Conference on Wireless Communications, Networking and Mobile Computing.

[6] Luo S., Luo L. Detection of an unknown frequency hopping signal based on image features. Proceedings of the 2nd International Congress on Image and Signal Processing.

[7] Weber J., Kowalske K., Robertson C., Kragh F., Brown C. Detection of frequency-hopped waveforms embedded in interference waveforms with noise. Proceedings of the 2nd International Congress on Image and Signal Processing.

[8] Javed F., Mahmood A. The use of time frequency analysis for spectrum sensing in cognitive radios. Proceedings of the 4th International Conference on Signal Processing and Communication Systems.

[9] Fargues M.P., Overdyk H.F., Hippenstiel R. Wavelet-based detection of frequency hopping signals. Proceedings of the Thirty-First Asilomar Conference on Signals, Systems and Computers.

[10] Sirotiya M., Banerjee A. Detection and estimation of frequency hopping signals using wavelet transform. Proceedings of the Second UK-India-IDRC International Workshop on Cognitive Wireless Systems.

[11] Overdyk H.F. (1997). Detection and estimation of frequency hopping signals using wavelet transforms. Master’s Thesis.

[12] Jaiswal K. Spectral sensing for cognitive radio: Estimation of adaptive frequency hopping signal. Proceedings of the IEEE Region 10 Conference.

[13] Jaiswal K. Spectral sensing of adaptive frequency hopping signal for cognitive radio. Proceedings of the IEEE International Performance, Computing and Communications Conference.

[14] Hampton J., Oetting J. A frequency hopping sequential detection technique for in-net coarse acquisition. Proceedings of the IEEE Millitery Communications Conference.

[15] Fan H., Guo Y., Xu Y. A novel algorithm of blind detection of frequency hopping signal based on second-order cyclostationarity. Proceedings of the Congress on Image and Signal Processing.

[16] Polydoros A., Woo K. (1985). LPI detection of frequency-hopping signals using autocorrelation techniques. IEEE J. Sel. Areas Commun..

[17] Chung C.D. Generalized likelihood-ratio detection of multiple-hop frequency-hopping signals. Proceedings of the IEEE Military Communications Conference.

[18] Dillard R.A., Dillard G.M. (1996). Likelihood-ratio detection of frequency-hopped signals. IEEE Trans. Aerosp. Electron. Syst..

[19] Levitt B.K., Cheng U., Polydoros A., Simon M.K. (1994). Optimum detection of slow frequency-hopped signals. IEEE Trans. Commun..

[20] Levitt B., Simon M., Polydoros A., Cheng U. Partial-band detection of frequency-hopped signals. Proceedings of the IEEE Global Telecommunications Conference.

[21] Snelling W.E. (1989). and Geraniotis, E. Sequential detection of unknown frequency-hopped waveforms. IEEE J. Sel. Areas Commun..

[22] Dillard R.A. (1979). Detectability of spread-spectrum signals. IEEE Trans. Aerosp. Electron. Syst..

[23] Miller L.E., Lee J.S., Torrieri D.J. (1993). Frequency-hopping signal detection using partial band coverage. IEEE Trans. Aerosp. Electron. Syst..

[24] Li H., Hu Y., Wang S. (2021). A novel blind signal detector based on the entropy of the power spectrum subband energy ratio. Entropy.

[25] Stoops S.A., Norman D., Prescott G.E. Simulation of radiometers for detection of spread spectrum signals. Proceedings of the Tactical Communications.

[26] Lehtomaki J.J., Juntti M. (2005). Detection of frequency hopping signals using a sweeping channelized radiometer. Signal Process..

[27] Lehtomaki J.J., Juntti M., Saarnisaari H. Detection of frequency hopping signals with a sweeping channelized radiometer. Proceedings of the Thirty-Eighth Asilomar Conference on Signals, Systems and Computers.

[28] Joo J., Won J., Lee C., Park S., Lee K. Detection of an unknown FH signal using scanning receiver and DF receiver in practical environments. Proceedings of the IEEE Wireless Communications and Networking Conference.

[29] Song M., Wigginton S. Frequency hopping pattern detection in wireless ad hoc networks. Proceedings of the IEEE Wireless Communications and Networking Conference.

[30] Donoho D.L. (2006). Compressed sensing. IEEE Trans. Inf. Theory.

[31] Candes E.J., Romberg J., Tao T. (2006). Robust uncertainty principles: Exact signal reconstruction from highly incomplete frequency information. IEEE Trans. Inf. Theory.

[32] Duarte M.F., Davenport M.A., Wakin M.B., Baraniuk R.G. Sparse signal detection from incoherent projections. Proceedings of the IEEE International Conference on Acoustics Speech and Signal Processing.

[33] Paredes J.L., Wang Z., Arce G.R., Sadler B.M. Compressive matched subspace detection. Proceedings of the 17th European Signal Processing Conference.

[34] Davenport M.A., Boufounos P.T., Wakin M.B., Baraniuk R.G. (2010). Signal processing with compressive measurements. IEEE J. Sel. Top. Signal Process..

[35] Zou J., Li Y., Dai W. (2013). Compressive detection with sparse random projections. IEICE Commun. Exp..

[36] Tropp J.A., Gilbert A.C. (2007). Signal recovery from random measurements via orthogonal matching pursuit. IEEE Trans. Inf. Theory.

[37] Wu J., Liu N., Zhang Y., Shen C. Blind detection of frequency hopping signal based on compressive sensing. Proceedings of the 2nd International Conference on Consumer Electronics, Communications and Networks.

[38] Liu F., Marcellin M.W., Goodman N.A., Bilgin A. Compressive detection of multiple frequency-hopping spread spectrum signals. Proceedings of the Data Compression Conference.

[39] Liu F., Marcellin M.W., Bilgin A., Goodman N.A. (2016). Compressive sampling for detection of frequency-hopping spread spectrum signals. IEEE Trans. Signal Process..

[40] Wang L., Chen M., Rodrigues M., Wilcox D., Calderbank R., Carin L. (2017). Information-theoretic compressive measurement design. IEEE Trans. Pattern Anal. Mach. Intell..

[41] Gu Y., Goodman N.A. Information-theoretic compressive measurement for frequency hopping pattern recognition. Proceedings of the 2018 Radar Conference (RadarConf18).

[42] Wang H., Guo D., Zhang B. Compressive sampling for recognition of frequency-hopping spread spectrum signals. Proceedings of the 2020 International Conference on Wireless Communications and Signal Processing (WCSP).

[43] Chong M., Li Q., Li J. (2019). Parameter estimation via deep learning for camera localization. IOP Conf. Ser. Mater. Sci. Eng..

[44] Gajecki T., Nogueira W. An end-to-end deep learning speech coding and denoising strategy for cochlear implants. Proceedings of the 2022 IEEE International Conference on Acoustics, Speech and Signal Processing (ICASSP).

[45] Xu D., Lu G., Yang R., Timofte R. Learned image and video compression with deep neural networks. Proceedings of the 2020 IEEE International Conference on Visual Communications and Image Processing (VCIP).

[46] Zhang J., Wu W., Huang J., Huang Y., Wang W., Su Y., Lyu M.R. Improving adversarial transferability via neuron attribution-based attacks. Proceedings of the 2022 IEEE/CVF Conference on Computer Vision and Pattern Recognition (CVPR).

[47] Li Z., Liu R., Lin X., Shi H. Detection of frequency-hopping signals based on deep neural networks. Proceedings of the IEEE 3rd International Conference on Communication and Information Systems.

[48] Lee K., Oh S. (2020). Detection of frequency-hopping signals with deep learning. IEEE Commun. Lett..

[49] Neifeld M.A., Ashok A., Baheti P.K. (2007). Task-specific information for imaging system analysis. J. Opt. Soc. Am. A.

[50] Specification of the Bluetooth System. http://grouper.ieee.org/groups/802/15/Bluetooth/profile_10_b.pdf.

[51] Arias-Castro E., Eldar Y.C. (2011). Noise folding in compressed sensing. IEEE Signal Process. Lett..

[52] Gu Y., Goodman N.A., Ashok A. (2014). Radar target profiling and recognition based on TSI-optimized compressive sensing kernel. IEEE Trans. Signal Process..

[53] Duarte-Carvajalino J.M., Yu G., Carin L., Sapiro G. (2013). Task-driven adaptive statistical compressive sensing of Gaussian mixture models. IEEE Trans. Signal Process..

[54] Yu G., Sapiro G. Statistical compressive sensing of Gaussian mixture models. Proceedings of the IEEE International Conference on Acoustics, Speech and Signal Processing.

[55] Tensorflow Software. https://www.tensorflow.org/.

